# Synthesis and Anticancer Activity of CDDO and CDDO-Me, Two Derivatives of Natural Triterpenoids

**DOI:** 10.3390/molecules24224097

**Published:** 2019-11-13

**Authors:** Rebecca Borella, Luca Forti, Lara Gibellini, Anna De Gaetano, Sara De Biasi, Milena Nasi, Andrea Cossarizza, Marcello Pinti

**Affiliations:** 1 Department of Life Sciences, University of Modena and Reggio Emilia, 41125 Modena, Italy; rebeccaborella7993@gmail.com (R.B.); luca.forti@unimore.it (L.F.); anna.degaetano@unimore.it (A.D.G.); 2 Department of Medical and Surgical Sciences of Children and Adults, University of Modena and Reggio Emilia, 41125 Modena, Italy; lara.gibellini@unimore.it (L.G.); debiasisara@yahoo.it (S.D.B.); 3 Department of Surgery, Medicine, Dentistry and Morphological Sciences, University of Modena and Reggio Emilia, 41125 Modena, Italy; milena.nasi@unimore.it (M.N.); andrea.cossarizza@unimore.it (A.C.)

**Keywords:** triterpenoids, bardoxolone methyl, anticancer drug, mitochondria

## Abstract

Triterpenoids are natural compounds synthesized by plants through cyclization of squalene, known for their weak anti-inflammatory activity. 2-cyano-3,12-dioxooleana-1,9(11)-dien-28-oic acid (CDDO), and its C28 modified derivative, methyl-ester (CDDO-Me, also known as bardoxolone methyl), are two synthetic derivatives of oleanolic acid, synthesized more than 20 years ago, in an attempt to enhance the anti-inflammatory behavior of the natural compound. These molecules have been extensively investigated for their strong ability to exert antiproliferative, antiangiogenic, and antimetastatic activities, and to induce apoptosis and differentiation in cancer cells. Here, we discuss the chemical properties of natural triterpenoids, the pathways of synthesis and the biological effects of CDDO and its derivative CDDO-Me. At nanomolar doses, CDDO and CDDO-Me have been shown to protect cells and tissues from oxidative stress by increasing the transcriptional activity of the nuclear factor (erythroid-derived 2)-like 2 (Nrf2). At doses higher than 100 nM, CDDO and CDDO-Me are able to modulate the differentiation of a variety of cell types, both tumor cell lines or primary culture cell, while at micromolar doses these compounds exert an anticancer effect in multiple manners; by inducing extrinsic or intrinsic apoptotic pathways, or autophagic cell death, by inhibiting telomerase activity, by disrupting mitochondrial functions through Lon protease inhibition, and by blocking the deubiquitylating enzyme USP7. CDDO-Me demonstrated its efficacy as anticancer drugs in different mouse models, and versus several types of cancer. Several clinical trials have been started in humans for evaluating CDDO-Me efficacy as anticancer and anti-inflammatory drug; despite promising results, significant increase in heart failure events represented an obstacle for the clinical use of CDDO-Me.

## 1. Introduction

Triterpenoids are natural compounds synthesized by plants through cyclization of squalene and represent one of the most numerous and diverse group of secondary metabolites, ubiquitously distributed in the plant kingdom [1]. So far, more than 20,000 natural triterpenoids are known [2], predominantly found in several medicinal plants, in wax-like coatings of various fruits such as apples, and in herbs including rosemary, oregano, and thyme [3,4,5]. The biological significance of these molecules is not completely clear: if the antibacterial and antifungal properties of some of them, such as oleananes, can explain their accumulation in fruits, most of these compounds present in plants are not toxic for herbivores or omnivores, and so have no obvious deterrent effect.

For centuries, extracts containing triterpenoids—which resemble steroids in chemical structure—have been used in Asian countries for medical purposes, as antibacterial, antifungal, antiviral, anti-inflammatory, antioxidant, antidiabetic, and hepato- and cardio-protective agents [3]. Oleanolic acid, one of these naturally-occurring triterpenoids, has a mild anti-inflammatory effect and a weak antitumorigenic activity [6,7,8]. However, most of the natural triterpenoids, including oleanolic acid, display their pharmacological activity at high concentration, up to 40 uM [9,10,11]. In an attempt to improve and enhance the biological activity of natural triterpenoids, a series of chemical modifications have been introduced to the structure of the molecules, and synthetic triterpenoids derived from oleanolic acid and ursolic acid have been obtained that exhibit optimized bioactivity such as potent anti-inflammatory and antitumorigenic activities [12]. In particular, 2-cyano-3,12-dioxooleana-1,9(11)-dien-28-oic acid (CDDO), and its C28 modified derivatives, methyl-ester (CDDO-Me, also known as bardoxolone methyl), and imidazole (CDDO-Im) have been extensively investigated for their strong antiproliferative, antiangiogenic, antimetastatic activities, and for their capability to induce apoptosis and differentiation in cancer cells. Here, we discuss the chemical properties of natural triterpenoids, the pathways of synthesis and the biological effects of CDDO and its derivative CDDO-Me.

## 2. Chemical Properties and Synthesis Pathways of Triterpenoids

Triterpenoids present a carbon skeleton containing six isoprene units, which are derived from the acyclic C30 hydrocarbon squalene, an important precursor for synthesis of all plant and animal sterols, including cholesterol and steroid hormones in humans.

**Figure 1:**
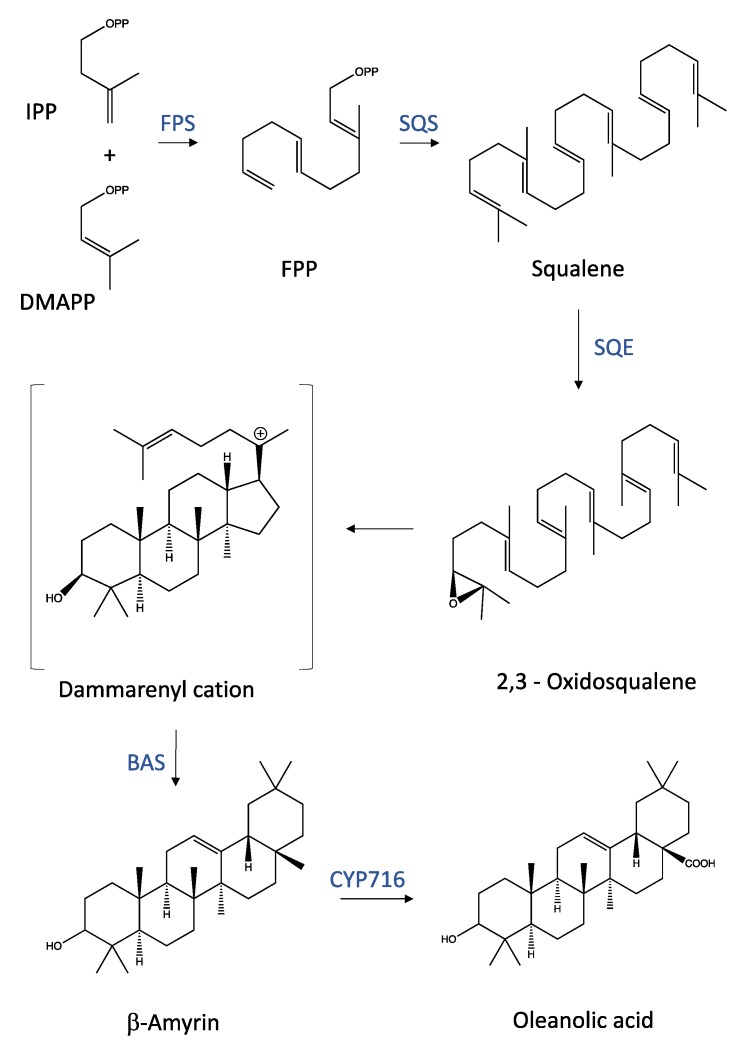
Synthesis pathway in plants and structure of oleanolic acid. See text for details. Abbreviations: DMAPP, dimethylallyl diphosphate; IPP, isopentenyl diphosphate; FPP, farnesyl pyrophosphate; FPS, farnesyl pyrophosphate synthase; SQS, squalene synthase; SQE, squalene epoxidase: BAS, β-amyrin synthase; CYP716, P450 enzymes belonging to CP71 group (CP716A12, CP716A15, CP716A17, CP716AL1). Enzyme names are in blue.

In plants, terpenoids share a common biosynthetic origin: all terpenoids derive from the repetitive fusion of isoprene (C_5_H_8_) units, and the number of isoprene units determines their classification. First, an isopentenyl pyrophosphate (IPP) is generated by the mevalonate (MVA)/3-hydroxy-3-methylglutaryl-CoA reductase (HMGR) pathway or the 2-*C*-methyl-d-erythritol 4-phosphate (MEP)/1-deoxy-d-xylulose 5-phosphate (DOXP)/non-MVA pathway. Then, the IPP is isomerized to dimethylallyl pyrophosphate (DMAPP), and the condensation of IPP and DMAPP units, catalyzed by specific prenyltransferases, forms prenylated pyrophosphates, the precursors of different terpenoid classes. Terpenoid synthases modify these precursors to terpenoid skeletons [13], which are further modified to different terpenoids [14]. Synthesis of triterpenoids is obtained through a condensation of two IPP units with a DMAPP unit, which generates the C_15_ farnesyl pyrophosphate (FPP). Two FPP are fused ‘head-to-head’ to generate squalene, the linear C_30_ precursor of triterpenoids. Then, squalene is epoxidized to 2,3-oxidosqualene [15] and cyclized to tetra- or pentacyclic structures by specific oxidosqualene cyclases (Figure 1). Oleanolic acid, one of these pentacyclic triterpenoids, is the precursor of several synthetic or semisynthetic compounds, including CDDO, CDDO-Me, and CDDO-Im [1].

**Figure 2:**
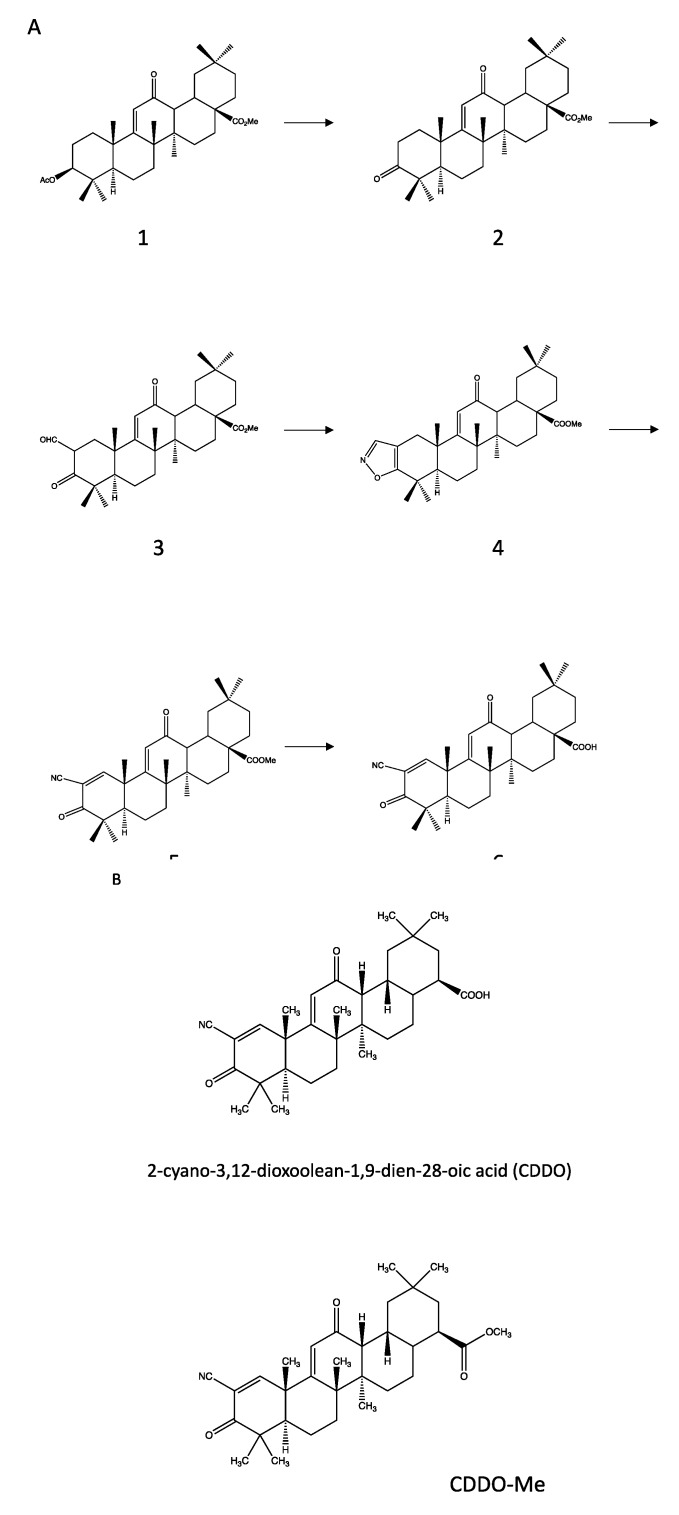
Structures and synthesis of CDDO and CDDO-Me, two synthetic derivatives of oleanolic acid. (**A**) Synthesis pathway of CDDO, as described by Honda et al. [18]. Compound **2** was obtained from the already known compound **1** by alkali hydrolysis and Jones oxidation. Compound **3** was obtained by formylation of **2** with ethyl formate; compound **4** was obtained from **3** by addition of hydroxylamine, and compound **5** by cleavage of isoxazole 16 with sodium methoxide and subsequent double bond introduction at C1 with PhSeC1–H_2_O_2_. CDDO (compound **6**) was obtained from **5** by halogenolysis of **5** with lithium iodide in dimethylformamide. (**B**) Structure of CDDO and CDDO-Me. CDDO-Me is the C28 methyl ester of CDDO.

Total synthesis of naturally occurring pentacyclic triterpenes, which have at least eight chiral centers, and in particular of oleanolic acid, has been at the center of intense research activity for decades. The first enantioselective total synthesis of oleanolic acid was reported in 1993 [16]. Oleanolic acid derivatives were firstly synthesized in 1998, in an attempt to identify new inhibitors of nitric oxide (NO) production in macrophages [17]. Honda et al. randomly modified the structure of oleanolic acid and obtained about 60 compounds, which were tested in vitro as inhibitors of the production of NO induced by IFN-γ. Several compounds showed significant inhibitory activity and one of them (3,12-dioxoolean-1,9-dien-28-oic acid) displayed the highest activity (IC_50_ = 7 μM) [17]. In a following study, the same research group reported the synthesis of a much more potent compound, named CDDO, whose inhibitory activity was comparable to that of dexamethasone [18]. A further efficient and multi-gram level synthesis procedure of CDDO-Me was described in 2013 [19]. The synthesis pathway of CDDO, as described in [18] is illustrated in the Figure 2A; Figure 2B shows the structure of CDDO and of its methyl derivative, CDDO-Me.

## 3. Anticancer Effects of Triterpenoids

Triterpenoids have pleiotropic effects. At low doses they display anti-inflammatory, antioxidative stress effects, while at intermediate doses they are able to induce cell differentiation and at high doses they exert cytotoxic, antiproliferative, and proapoptotic effects. Thus, they can theoretically have an anticancer activity at multiple levels: the low doses of these molecules can prevent the process of carcinogenesis and mitigate the damage of procarcinogens, while at intermediate–high levels they can slow down the proliferation of cancer cells, and/or cause cell death by apoptosis.

The antiproliferative activity of CDDO-Me is generally observed at concentrations ranging from 0.1 to 1.0 μM, and the proapoptotic activity can be seen with concentrations above 0.5 μM. CDDO is slightly less effective than CDDO-Me and is active at concentrations 5–10 times higher.

As far as antiproliferative effect is concerned, the most striking features of these molecules are their capability to inhibit cell growth independently from p53 status, and the fact that inhibition is often observed in neoplastic cells, but not in their normal counterpart. The latter point has particularly drawn the attention of researchers for using CDDO and its derivatives in cancer treatment.

It has been proposed that the effects of CDDO and CDDO-Me are largely mediated by the presence of two electrophilic Michael acceptor sites in the A and C rings, which allow the formation of adducts with proteins containing redox-sensitive Cys residues; this effect has been directly demonstrated in the case of relevant targets such as the IκB kinase (IKK), the ubiquitin-specific-processing protease 7 (USP7) or the erythroblastic oncogene B2 (ErbB2) [20,21,22,23]. All these proteins contain specific Cys crucial for their functions, and CDDO and CDDO-Me interact only with some of them, in a specific manner. This mechanism of action has been directly proved in the case of IKK: the Cys179 is specifically targeted in this protein, and when this residue is mutated, the effect of CDDO-Me on NF-κB pathway is abrogated.

Thus, different concentrations of CDDO and CDDO-Me can have different—even opposite—effects on target cells because of the diverse binding affinity for target proteins when forming Michael adducts. At low concentrations, these synthetic triterpenoids interact with kelch-like ECH-associated protein 1 (Keap1) and activate a cytoprotective pathway, while at higher concentrations other proteins (such as PPAR-γ, USP7, IKK, Lonp1) are targeted to inhibit proliferation or induce apoptosis. It must be noted that the formation of Michael adducts by CDDO and CDDO-Me is reversible. Thus, it is possible that CDDO and CDDO-Me induce a biological response in a cell by binding a specific target, but they may not remain bound to this target, making the effect transient.

### 3.1. Effects In Vitro at Low Doses

At nanomolar concentrations, CDDO and CDDO-Me have been shown to protect cells and tissues from oxidative stress by increasing the transcriptional activity of the nuclear factor (erythroid-derived 2)-like 2 (Nrf2). Nrf2 is the principal regulator of the phase II cellular antioxidant response and represents an endogenous defense mechanism against toxic cell stress [24]. Under physiological conditions, Nrf2 is sequestered in the cytosol by its repressor protein, Keap1, and is subsequently ubiquitinated and degraded [25,26]. During cell stress, ubiquitination of Nrf2 by Keap1 is disrupted, allowing Nrf2 to translocate to the nucleus, and up-regulate genes containing an antioxidant response element (ARE) in their promoter regions [27]. Nrf2- regulated genes facilitate a variety of functions, including antioxidative activity, detoxification and transport of xenobiotics, proteasome activity, and well as glutathione homeostasis [28]. Electrophilic compounds have been shown to induce the activation of the Nrf2 pathway and many of them (including CDDO), have demonstrated cytoprotective responses against oxidative and inflammatory stress in vitro [29,30,31]. Along with the activation of the antioxidative response, nanomolar doses of CDDO and its derivatives have also an antioxidant effect. The mechanisms at the basis of this effect is not completely understood, but is at least in part due to the capability of these molecules to suppress iNOS in innate immune cells [18,32], and to reduce the expression of proinflammatory cytokines, including (but not limited to) tumor necrosis factor (TNF)-α, interleukin (IL)-1β, IL-6, and interferon (IFN)-γ in a variety of cell types [33,34,35,36,37,38,39,40]. It is interesting to note that CDDO-Me has an opposite effect in M2 macrophages, as it reduces anti-inflammatory cytokines like IL-10 and increases the production of TNF-α and IL-6 [40]. 

While the activation of Keap1/Nrf2/ARE pathway by CDDO and its derivatives has been shown to be beneficial in several experimental models of human diseases, such as neurodegenerative diseases [30,31,41,42,43,44], eye diseases [45,46], and lung pathologies [47,48], the lines of evidence of a possible effect on cancer development are less convincing. Indeed, several studies have provided evidence that CDDO, CDDO-Me can act as a chemopreventer in vivo (see Section 5) but few of them have shown a possible role of the Nrf2 pathway in this process. This is probably because in some tumor models, activation of the Nrf2 pathway does not prevent cancer development but can be detrimental by aiding carcinogenesis or giving rise to resistance to chemotherapeutic drugs. 

Synthetic triterpenoids can also have an antiproliferative and proapoptotic effect at these low doses. CDDO-me inhibits the activation of the PI3K/Akt/mTOR pathway, which is often dysregulated in cancer [49,50]. In human prostate cancer cells, CDDO-Me inhibits the activity of *p*-Akt and mTOR and of their downstream targets [51,52] and overexpression of Akt leads to resistance to CDDO-Me [52]. The inhibition of this pathway is likely ROS-dependent, as the inhibition of ROS generation by antioxidants like *N*-acetylcysteine (NAC) prevents the inhibition of constitutively active Akt, nuclear factor κB (NF-κB), and mTOR by CDDO-Me. A similar mechanism of action has been demonstrated in several other cancer cell lines, such as pancreatic cancer cells [53,54], colorectal cancer cells [55,56], ovarian cancer cells [57,58], human glioblastoma and neuroblastoma cell lines [55], suggesting that this is a general phenomenon.

### 3.2. Effects In Vitro at Intermediate Doses

At doses higher than those needed for activating Nrf2 pathways (>100 nM), CDDO and CDDO-Me are able to modulate the differentiation of a variety of cell types, both tumor cell lines and primary culture cells. This effect has been observed in human leukemia cell lines, monocytic cell lines, osteosarcoma and adipocytes, while an effect on stem cells or progenitor cells has been observed with CDDO-Im, but not with CDDO or CDDO-Me [59,60,61,62,63,64].

The mechanisms that underpin the induction of cell differentiation by the CDDO and CDDO-Me are far from being clear, and several factors involved in cell differentiation have been identified as targets of synthetic triterpenoid activity.

The most convincing data have been obtained in preadipocytes, where CDDO has been shown to induce adipocyte differentiation by modulating PPAR-γ activity. In these cells, CDDO shows a biphasic activity. When used at doses ranging from 10 to 100 nM, it induces differentiation of 3T3-L1 preadipocytes. At the dose of 1 uM, CDDO fails to induce differentiation, and inhibits that caused by all other known differentiating molecules, such as rosiglitazone. While the mechanism of inhibition is unknown, the differentiation effect is due to the binding of CDDO to PPAR-γ, which is an agonist. Unlike the cases of IKKβ, JAK, or STAT3, CDDO binds to PPAR-γ in a non-covalent and reversible manner, as no direct adduct formation has been observed. It is interesting to note that, even if CDDO-Me can bind to PPAR-γ with similar affinity, CDDO-Ma acts as an antagonist of the transactivator; this striking difference is due to the fact that CDDO releases the nuclear receptor corepressor (NCoR) from PPAR-γ, while CDDO-Me does not [65]. The involvement of PPAR-γ in the differentiation activity of synthetic triterpenoids has been independently confirmed in other cell models, such as the acute promyelocytic leukemia (AML) HL-60, NB4, and MR2 cell lines, and in patient-derived primary AML blasts. In HL-60 cells, CDDO induces PPAR-γ activation, and enhances binding of the vitamin D-interacting protein (DRIP205) coactivator to PPAR-γ. Accordingly, the PPAR-γ antagonist T007 blocks differentiation in HL-60 cells treated with CDDO, and the differentiation induced by CDDO is enhanced in the same cells overexpressing DRIP205 [66].

CDDO is able to enhance the differentiating effect of all-trans-retinoic acid (ATRA) in two models of acute promyelocytic leukemia (APL): in the ATRA-sensitive cell line NB4, CDDO enhances the differentiating effect of ATRA, while in the ATRA-resistant MR2 cell line it partially reverses ATRA resistance [63]. These effects are mediated by CDDO-induction of PPAR-γ, and by enhancing the ability of ATRA to induce retinoic acid receptor (RAR) β2 gene expression in APL cells. Independently from the effect on PPAR-γ , the combination of ATRA and CDDO partially increases histone acetylation in the RARβ2 promoter, which in turn allows the recruitment of RARE to the RARβ2 promoter [63]. 

CDDO is also able to induce differentiation in a PPAR-γ independent manner. In osteosarcoma cells, differentiation induced by CDDO is abrogated by the overexpression of the extrinsic caspase-8 inhibitor cytokine response modifier A (CrmA), suggesting that CDDO-induced differentiation is mediated by caspase-8 activity [67]. 

### 3.3. Effects In Vitro at High Doses

At high doses (about 1–10 μM, depending on the molecule and cell model) CCDO and its derivatives exert an anticancer activity through several mechanisms, often selective for proliferating cancer cells but not for non-transformed cells. Although most of the mechanisms described below act directly on cancer cells, CDDO and its derivatives can also redirect and reactivate the immune response versus cancer cells [40].

#### 3.3.1. Induction of Apoptosis

Apoptosis can be triggered via either the caspase-mediated extrinsic or intrinsic pathways [68]. In the extrinsic or death receptor mediated pathway, binding of death ligands (e.g., FasL, TRAIL) with their death receptors activates initiator caspase-8, which cleaves and activates effector caspases 3, 6, and 7, which ultimately lead to apoptosis [68]. The intrinsic apoptotic pathway is triggered by stimuli that trigger permeabilization of mitochondria and release of cytochrome-c (cyt-c) into the cytoplasm [69]. Cyt-c binds to Apaf-1 and forms the apoptosome, which in turn recruits and activates caspase-9, the first caspase of the intrinsic pathway effector caspases 3, 6, and 7 [68].

The literature discussing how CDDO and CDDO-Me trigger apoptosis is extensive, with notable discrepancies in the different cell models. As a general rule, CDDO has been shown to activate extrinsic apoptosis, while CDDO-Me activates the intrinsic pathway [67,70,71,72,73]. CDDO exerts its activity on extrinsic pathway at multiple levels. First, it activates caspase-8, which in turns leads to the activation of caspase-3 and, via cleavage of Bid, to the release of cytochrome c from mitochondria. Second, treatment with CDDO upregulates death receptors (DR) 4 and 5 and downregulates the antiapoptotic protein cellular FLICE-like inhibitory protein (c-FLIP) [74,75]. Conversely, convincing lines of evidence have been reported that CDDO-Me preferentially activates the intrinsic pathway, by upregulating the proapoptotic protein Bax, or by permeabilizing the inner mitochondrial membrane, so favoring cyt-c release [72,76]. Nevertheless, some models in which CDDO-Me activates the extrinsic pathway have been reported: by depleting GSH and causing reticulum stress, CDDO-Me activates c-Jun NH_2_-terminal kinase (JNK), which activates CCAAT/enhancer-binding protein homologous protein (CHOP). CHOP in turn upregulates decoy receptor (DR) 5, so favoring apoptosis [77,78].

The mechanisms by which CDDO and CDDO-Me initiate the apoptotic cascade are far from being clear. One proposed mechanism is that these molecules disrupt the oxidative balance of cells, but it is difficult to establish if increase in cellular ROS is an initiating event, or if it is rather a consequence of mitochondrial perturbation [79]. CDDO and CDDO-Me decrease intracellular GSH levels [79], one of the most important scavengers of reactive oxygen species (ROS). The maintenance of correct GSH levels is important not only for scavenging ROS but also for preventing apoptosis triggered by mitochondria, which do not synthetize GSH but import it from the cytosol [80]. The cytotoxic effects of CDDOs are mediated by a rapid and selective decrease of mitochondrial GSH (mGSH) which leads to caspase-independent apoptosis [76]. In this model, CDDO-Im induced depletion of mGSH occurs prior to the onset of apoptosis and results in the generation of ROS, mitochondrial dysfunction, and intracellular glutathione pool oxidation. Treatment with the triterpenoid induced rapid alterations in the cytoplasmic morphology that were insensitive to the pharmacological inhibition of caspases. Similarly, the loss of mitochondrial membrane potential was also not prevented by the inhibition of caspases. Notably, cotreatment with sulfhydryl antioxidants prevents the depletion of mGSH, the loss of mitochondrial membrane potential, and the shrinking of cytoplasm, suggesting that redox stress can mediate the activation of caspase-independent apoptosis [76].

#### 3.3.2. Induction of Autophagy

Less attention has been paid to the effects of these compounds on autophagy. CDDO-Me is able to induce autophagy in chronic myeloid leukemia cells, in which the toxic effect on mitochondria is rapidly followed by engulfment in autophagosomes of damaged organelles or by mitochondrial-induced apoptosis [81]. In K562 cells, CDDO-Me induces autophagy by suppressing the PI3K/Akt/mTOR signaling pathway [82]. The same mechanism has been independently observed in esophageal squamous cancer cell lines Ec109 and KYSE70, suggesting that this effect of CDDO-Me could be a general phenomenon [83].

#### 3.3.3. Inhibition of Janus-Activated Kinases (JAKs)

Signal transducer and activator of transcription (STAT) proteins are involved in the regulation of cell proliferation and differentiation, and of apoptosis [84]. Among them, STAT3 is often constitutively active in cancer cells, and can contribute to neoplastic transformation, invasion, and metastasis. After binding of a ligand to its cognate growth factor receptor, such as the IL-6 receptor, a JAK kinase phosphorylates the receptor, allowing recruitment and phosphorylation of a STAT. STATs then dimerize, translocate to the nucleus, and induce the transcription of several STAT targets, including cyclin D1, myc, and survivin proteins [85,86]. CDDO-Me has been shown to inhibit JAK/STAT3 pathways in different cell models, such as in ovarian cancer breast cancer and osteosarcoma cancer cells [37,87,88]. Studies on the mechanisms of action demonstrated that CDDO-Me inhibits this pathway at multiple levels and in a specific way. At micromolar concentrations, CDDO-Me suppresses JAK1 phosphorylation by direct binding to Cys1077. Thus, Jak1 is unable to phosphorylate STAT3, which is crucial for its dimerization and activation [87]. Independently from the effect on Jak1, CDDO-Me can also form adducts with STAT3 that are dependent on Cys259, as mutations of this aminoacidic residue abrogates the inhibitory effect [87].

#### 3.3.4. Inhibition of NF-κB Activity

Synthetic triterpenoids, and in particular CDDO-Me, can exert their activity also by inhibiting the NF-κB pathway. NF-κB is a transcription factor crucial for activating a variety of genes involved in inflammation, proliferation, and survival. Since the proinflammatory microenvironment is a feature of most cancers, its inhibition can play a role in inhibiting tumorigenesis and cancer proliferation. This capability has been shown in a variety of cells, including PC-3 and C4-2 cells, prostate cancer cells, and colorectal cancer cells, where CDDO-Me inhibits the growth and causes cells death at intermediate–high concentrations (0.625–2.5 μM) through the inhibition of NF-κB, p-Akt, and mTOR [52].

#### 3.3.5. Inhibition of Telomerase Activity

CDDO-Me can inhibit cancer proliferation and provoke apoptosis by inhibiting telomerase activity, which is associated with promotion of tumorigenesis [53]. When telomeres, which shorten at any cell division, becomes too short, they trigger senescence or apoptosis. The enzyme telomerase can counteract this process by elongating telomeres and cancer cells often upregulate telomerase to circumvent cell senescence due to telomere shortening. In a model of human pancreatic cancer cells, CDDO-Me is able to inhibit telomerase gene and protein expression, as well as its enzymatic activity [54], through a ROS-dependent mechanism [89]. The action of CDDO-Me on telomerase activity is also indirect, as it inhibits c-Myc, Sp1, NF-κB, p-STAT3, and p-Akt, a group of factors regulating telomerase, and causes decreased histone deacetylation and histone demethylation at the promoter of the human telomerase gene [90]. This inhibitory effect on telomerase is likely a general phenomenon, as it has been demonstrated in other cancer cell lines [58,91].

#### 3.3.6. Inhibition of Mitochondrial Protease Lonp1

More recently, we and others have shown that CCDO and CDDO-Me can exert their antiproliferative, proapoptotic activity by inhibiting the mitochondrial protease Lonp1. This is a ubiquitous serine protease present in the mitochondrial matrix, but encoded by the nucleus, which has three main functions: (i) it degrades oxidized or damaged proteins, with an ATP-dependent mechanism; (ii) it acts as a chaperone for the folding of imported proteins into the mitochondrial matrix; (iii) it binds mtDNA and contributes to the maintenance of the normal levels of this molecule in the mitochondria [92,93,94]. Lonp1 plays a critical role in antioxidant stress response acting as a chaperone for the degradation of mutant and abnormal proteins, such as toxic aggregates of oxidized mitochondrial proteins, and regulates the maintenance of mitochondria DNA, morphology, and dynamics [93]. CDDO and its derivatives mediate apoptosis in lymphoma cells through a mitochondria-mediated mechanism, by which CDDO leads to mitochondrial protein thiol modification and the generation of mitochondrial protein aggregates, which in turn contribute to the increase in the permeability of mitochondrial membranes and lead to the initiation of apoptosis [95]. The CDDO-induced mitochondrial protein aggregation can be the consequence of the inhibition of the mitochondrial ATP-dependent Lon protease. Then, CDDO and its derivatives have been demonstrated to directly and selectively inhibit Lonp1: CDDO blocks Lonp1-mediated proteolysis in biochemical and cellular assays but does not inhibit the 20S proteasome. Furthermore, a biotinylated-CDDO conjugate modifies mitochondrial Lonp1. As Lonp1 protein levels are increased in malignant lymphoma cells if compared with B cells, and considering that Lonp1 knockdown causes lymphoma cell death, the pharmacological inhibition of Lonp1 by CDDO could represent a promising therapeutic approach for B-cell lymphoma [95]. Our group expanded and deepened such idea. Using colon cancer cellular models, we observed that CDDO and CDDO-Me decrease proliferation and induce apoptosis in a dose-dependent manner. Furthermore, they are able to determine an increase in mitochondrial hydrogen peroxide and mitochondrial superoxide anion, which in turn induces the increase of carbonylation of mitochondrial proteins, causing mitochondrial depolarization, reduction of mitochondrial mass, and alteration of the organelle morphology [96]. In particular, both molecules determine an evident fragmentation of the mitochondria and the loss of the normal morphology of the matrix and of the cristae. This effect is due, at least in part, to the inhibition of Lonp1 functions, since the expression of this enzyme is not significantly altered by CDDO and CDDO-Me, but the levels of Lonp1 enzymatic activity targets, such as aconitase or TFAM, significantly increase. In line with these observations, Lonp1 overexpression abrogates the effects of CDDO and CDDO-Me, protecting cells from apoptosis [97,98].

#### 3.3.7. Inhibition of Ubiquitin-Specific-Processing Protease 7 (USP7)

The last mechanism demonstrated for the anticancer activity of CDDO-Me is the inhibition of USP7, a deubiquitylating enzyme that cleaves ubiquitin from its substrates [22]. USP7 is the antagonist of MDM2, the regulator of p53 levels, and is involved in the pathogenesis and progression of several types of cancers. CDDO-Me directly binds to USP7 in cells, likely in its ubiquitin carboxyl terminus-binding pocket, and inhibits its activity, leading to the decrease of its substrates MDM2, MDMX, and UHRF1. This effect has been demonstrated in an in vitro model of ovarian cancer, and further confirmed by suppression of tumor growth in a xenograft model. 

The anticancer effects described in the following paragraphs are summarized in Table 1. 

## 4. Anticancer Effects In Vivo

Several studies have been performed to test if CDDO and CDDO-Me anticancer effects observed in vitro could be mirrored by a similar effect in animal models. Most of the studies in animal models have been focused on CDDO-Me, which was considered the most potent molecule, and the most promising as a candidate for tests in humans. CDDO-Me demonstrated its efficacy as anticancer drugs in different mouse models, and versus several types of cancer. Doses of CDDO-Me tested were between 7.5–60 mg/kg/day (see Table 2).

CDDO-Me has been shown to inhibit lung carcinogenesis in vivo. Treatment with vinyl carbamate, a potent mutagenic agent, induces lung adenocarcinoma in female A/J mice in 16 weeks, but treatment with CDDO-Me together with vinyl carbamate markedly reduced number, size, and severity of tumors [99]. The same group observed that in another model of carcinoma, i.e., the mouse mammary tumor MMTV-neu transgenic model, CDDO-Me plus the rexinoid LG100268 significantly delayed the onset of estrogen receptor (ER)-negative mammary tumors if compared to controls [100]. The effects of the two drugs were synergic, as mice treated with both compounds showed a much higher reduction of tumor development than mice treated with individual drugs.

CDDO-Me also delays mammary carcinogenesis in the aggressive PyMT model of estrogen receptor-negative breast cancer. In this model, the PyMT gene is under the control of the MMTV promoter, and the mice developed a tumor that recapitulates the key features of the human disease [101]. CDDO-Me, at the dose of 50 mg/kg/day, significantly delays tumor onset. This increase in survival is mediated by different mechanisms: inhibition of EGFR and STAT3 pathways, reduction in the infiltration of tumor-associated macrophages in the tumor microenvironment, reduction of levels of chemokines able to attract and activate lymphocytes and monocytes, such as CXCL12 and CCL2, and decreased secretion of matrix metalloproteinases, crucial for invasion and metastasis [102]. 

CDDO-Me delays tumor development in a mouse model with ablation of breast cancer-associated gene (BRCA1) and single allele mutation of p53 (Brca1Co/Co; MMTV-Cre; p53^+/−^ mice). In this model, supplementation of CDDO-Me in the diet from 12 weeks of age delayed breast cancer development by an average of 5.2 weeks [23]. 

CDDO-Me inhibits the progression of preneoplastic lesions to adenocarcinoma in a transgenic mouse model of prostate adenocarcinoma [103]. The delayed progression has been observed in more than 70% of the mice and importantly, no evident toxicity of the drug was observed [103]. Not surprisingly, studies on primary cell culture from the same model showed that the anticancer effect was due to antiproliferative, proapoptotic effect of CDDO-Me, mediated by the downregulation of Akt, mTOR, NF-κB, and of the NF-κB-regulated antiapoptotic and proangiogenic proteins [104], as well as to the reduction of telomerase reverse transcriptase activity [91]. A similar effect has been observed in a transgenic model of pancreatic cancer that recapitulates the genetic mutations, clinical symptoms, and histopathology of the human disease [105]. In this model, CDDO-Me, alone or in combination with the drug LG268, increases survival of mice by 3–4 weeks. 

Finally, CDDO-Me enhances the efficacy of vaccine therapy for melanoma [106]. In an experimental model of melanoma, the efficacy of Trp2 vaccination in female C57BL/6 mice inoculated with inoculated with B16F10 melanoma cells was significantly increased by CDDO-Me. The enhanced efficacy of the vaccine is due to the remodeling of the tumor microenvironment, in which CDDO-Me, delivered by nanoparticle to the tumor mass, remodels the tumor-associated fibroblasts, collagen and vessel and enhances the Fas signaling pathway, which in turn sensitizes cancer cells for killing by cytotoxic T lymphocytes. 

## 5. Anticancer Effects in Humans: Clinical Trials

Since pre-clinical studies showed beneficial activity of CDDO-Me in animal models as an antitumor compound, several clinical trials have been conducted in humans to test its efficacy to evaluate its activity against solid tumors and lymphoid malignancies. In these trials, CDDO-Me is usually referred to as bardoxolone methyl or RTA-4012. So far, 33 clinical trials have been registered in clinicaltrials.gov.

The first phase I clinical trial of CDDO-Me was conducted in patients with advanced solid tumor and lymphoma to identify the determine the dose-limiting toxicity (DLT) and the maximum tolerated dose (MTD). CDDO-Me was administered orally once a day for 21 days. The MTD was established as 900 mg/day and was associated with the antitumor activity, with complete response in a patient with mantle cell lymphoma, and partial response in a patient with anaplastic thyroid carcinoma [107]. In this first trial, an increase in estimated glomerular filtration rate (eGFR) was also noted. This observation led to the proposal to use CDDO-Me for treatment of patients with chronic kidney disease (CKD) and prompted a phase II trial in patients with moderate to severe CKD and type 2 diabetes. In this trial, patients received placebo or oral CDDO-Me at a dose of 25, 75, or 150 mg once daily for 52 weeks. Kidney function improvements were observed, and only mild to moderate adverse effects occurred, with muscle spasms, hypomagnesemia, mild elevations in alanine aminotransferase levels, and gastrointestinal effects being the most common [108]. 

Then, a phase III trial, named BEACON (NCT01351675) was designed to test the efficacy of CDDO-Me on patients with stage 4 CKD and type 2 diabetes [109,110]. BEACON was a randomized, double-blind, parallel-group, international, multicenter trial of once-daily administration of 20 mg of CDDO-Me, compared with placebo. Patients enrolled in BEACON were adults with T2DM and stage 4 CKD. Patients received background conventional therapy (inhibitors of the renin-angiotensin-aldosterone system, insulin or hypoglycemic agents) and were randomized 1:1 for administration of CDDO-Me or placebo. BEACON was stopped early because of a significant increase in heart failure events within the first 4 weeks of treatment [109]. These events were caused by fluid retention and occurred in patients with prior history of heart failure and elevated baseline B-type natriuretic peptide, while no evidence of direct cardiotoxicity was observed [111,112]. Thus, trials are ongoing, focused on the use of CDDO-Me for treating CKD or pulmonary hypertension, rather than for cancer treatment.

## 6. Conclusions and Future Perspectives

CDDO and CDDO-Me represent interesting examples of molecules derived from natural compounds that potentiate the effects of the natural counterpart. Nevertheless, the difficulty to identify all the targets and mechanisms of action of these compounds, as well as the toxic effects observed in clinical trials limit their potential as candidates for cancer treatment in humans. As the potential of natural terpenoids remains largely unexplored, it is likely that other triterpenoids and derivatives hold potential as future therapeutics. 

## Figures and Tables

**Table 1:** In vitro evidence of CDDO and CDDO-Me anticancer activity.

Compound	Cell Line(s)	Effect(s)	Reference(s)
**CDDO-Me**	LNCaP, DU145, and PC3 prostate cancer cell lines	Inhibition of proliferation and induction of apoptosis; Inhibition of Akt, mTOR, NF-κB, and NF-κB-regulated antiapoptotic and proangiogenic proteins	[51]
**CDDO-Me**	PC-3 (AR(–)) and C4-2 (AR(+)) prostate cancer cells	Growth inhibition and induction of apoptosis; Inhibition of p-AKT and mTOR	[52]
**CDDO-Me**	MiaPaCa-2 and Panc-1 pancreatic cancer cells	Downregulation of p-Akt, p-mTOR and NF-kappaB; Generation of hydrogen peroxide and superoxide anion	[54]
**CDDO-Me**	U87MG, U251MG glioblastoma, and SK-N-MC neuroblastoma cell lines	Inhibition of antiapoptotic and prosurvival p-Akt, NF-kappaB (p65), and Notch1 molecules; Induction of apoptosis	[55]
**CDDO**	U87MG, U251MG glioblastoma and SK-N-MC neuroblastoma cell lines	Induction of apoptosis	[55]
**CDDO-Me**	HCT 8, HCT-15, HT-29, and Colo 205 colorectal cancer cells	Growth inhibition and induction of apoptosis; Generation of reactive oxygen species; Inhibition of Akt, mTOR, and NF-κB	[56]
**CDDO-Me**	OVCAR-3, OVCAR-5, and SK-OV3 ovarian cancer cell lines	Growth inhibition and induction of apoptosis; Inhibition of p-AKT, NF-κB, and p-mTOR	[57]
**CDDO-Me**	OVCAR-5 and MDAH 2774 ovarian cancer cells	Growth inhibition and induction of apoptosis; Inhibition of p-AKT, NF-κB, and p-mTOR; Inhibition of BCL-2, BCL-xL, c-IAP1	[58]
**CDDO-Me**	Saos-2 osteosarcoma cells	Osteoblastic differentiation; Induction of apoptosis by caspase-8-dependent mechanisms	[67]
**CDDO-Me**	H460, A549, and H1944, H522, H157, and H1792 non-small-cell lung carcinoma cell lines	Induction of apoptosis via DR5 expression and caspase-8 activation	[69]
**CDDO-Me**	H460 and H1792 non-small-cell lung carcinoma cell lines	Trigger of ER stress; JNK-dependent, CHOP-mediated DR5 upregulation	[70]
**CDDO-Me**	H157 and A549 non-small-cell lung carcinoma cell lines	Induction of ubiquitin/proteasome-dependent c-FLIP degradation	[71]
**CDDO**	U-937 leukemia cells.	Induction of apoptosis via intrinsic pathway; Higher levels of ROS and lower levels of intracellular glutathione (GSH).	[73]
**CDDO-Me**	U-937 leukemia cells.	Induction of apoptosis via intrinsic pathway; Higher levels of ROS and lower levels of intracellular glutathione (GSH)	[73]
**CDDO-Me**	KBM5 chronic myeloid leukemia cells.	Induction of apoptosis and autophagic cell death	[76]
**CDDO-Me**	K562 chronic myeloid leukemia	Cell cycle arrest, apoptosis, and autophagy via PI3K/Akt/mTOR and p38 MAPK/Erk1/2	[77]
**CDDO-Me**	Ec109 and KYSE70 esophageal squamous cancer cells	Cell cycle arrest in G2/M phase; induction of apoptosis; Induction of autophagy by suppressing PI3K/Akt/mTOR pathway	[78]
**CDDO-Me**	MDA-MB-468 breast cancer cells	Inhibition of JAK1/STAT3 pathway	[82]
**CDDO-Me**	HeLa cervical cancer cells	Inhibition of JAK1/STAT3 pathway	[82]
**CDDO-Me**	KHOS, U-2OS, SaOS osteosarcoma cells	Induction of apoptosis via inhibition of STAT3 nuclear translocation and Bcl-X_L_, survivin, and MCL-1 downregulation	[83]
**CDDO-Me**	MiaPaCa-2 and Panc-1 pancreatic cancer cell lines	Inhibition of telomerase activity though a ROS-dependent mechanism; Decrease of histone deacetylation and histone demethylation at hTERT promoter	[84,85]
**CDDO-Me**	LNCaP and PC-3 prostate cancer cell lines	Inhibition of hTERT gene expression and of hTERT telomerase activity	[86]
**CDDO, CDDO-Me**	OCI-Ly7, OCI-Ly19, OCI-Ly3, and OCI-Ly1 diffuse large B-cell lymphoma cell lines	Inhibition of Lonp1 protease activity	[90]
**CDDO, CDDO-Me**	RKO colorectal cancer cells	Impairment of mitochondrial proteome and block of mitochondrial respiration via Lonp1 inhibition	[91,92,93]
**CDDO, CDDO-Me**	SKOV3, OVCAR3, A2780, A2780/CP70, and HeyC2 ovarian cancer cell lines	Inhibition of deubiquitinating enzyme USP7	[22]

**Table 2:** Anticancer effects of CDDO and CDDO-Me in vivo described in the text.

Compound	Animal Model	Treatment	Effect(s)	Reference(s)
**CDDO-Me**	Female A/Jm mice	Oral assumption; 40 mg/kg from the 8th week of age	CDDO-Me reduced number size and severity of lung carcinomas induced by vinyl carbamate; acts synergistically with the rexinoid LG100268	[94]
**CDDO -Me**	FVB/N-Tg(MMTVneu)202Mul/J female mice	Oral assumption; 60 mg/kg from the 10th week of age for up to 45 weeks	CDDO-Me delays development of ER-negative tumors of 14 weeks; acts synergistically with the rexinoid LG100268	[95]
**CDDO-Me**	FVB/N-Tg(MMTV-PyVT)634Mul/J mice	Oral assumption; 50 mg/kg	CDDO-Me delays mammary carcinogenesis in PyMT breast ER-negative cancer by 4.3 weeks	[97]
**CDDO-Me**	*Brca1^Co/Co^; MMTV-Cre;p53^+/−^* mice	Oral assumption; 50 mg/kg	CDDO-Me delays breast cancer development by an average of 5.2 weeks	[96]
**CDDO-Me**	C57BL/6-Tg(TRAMP)8247Ng/J mice	Oral assumption; 7.5 mg/kg from the 5th week of age; treatment for 7 or 20 weeks.	CDDO-Me inhibits the progression of the preneoplastic lesions to prostate adenocarcinoma; inhibits metastasis	[98,99]
**CDDO-Me**	*LSL-Kras^G12D/+^; LSL-Trp53^R127H/+^; Pdx-1-Cre* (KPC) mice	Oral assumption; 60 mg/kg from the 4th week of age	CDDO-Me increases mice survival by 3–4 weeks; acts synergistically with rexinoid LG268	[100]
**CDDO-Me**	Female C57BL/6 mice	Intravenous injections of CDDO-Me nanoparticles; intraperitoneal injections of CDDO-Me every other day (5 mg/kg)	CDDO-Me enhances efficacy of vaccine therapy for melanoma	[101]
